# Burden of alcohol and other substance use and correlates among undergraduate students at Busitema University in rural Eastern Uganda after COVID-19 lockdown

**DOI:** 10.1038/s41598-024-56861-1

**Published:** 2024-03-14

**Authors:** Joseph Kirabira, Enid Kawala Kagoya, Joseph Mpagi, Christine Etoko Atala, Kalisiti Ndamanywa, Ambrose Okibure, Ronald Kibuuka, Fauz Katongole, Julius Wandabwa

**Affiliations:** 1 Department of Psychiatry, Busitema University, Faculty of Health Sciences, P.O. Box 1460, Mbale, Uganda; 2 Institute of Public Health, Department of Community Health, Busitema University, Faculty of Health Sciences, P.O. Box 1460, Mbale, Uganda; 3 Deans Office, Department of Academics, Research and Innovation, Busitema University, Faculty of Health Sciences, P.O. Box 1460, Mbale, Uganda; 4 Department of Anaesthesia, Mbarara University of Science and Technology, P.O. Box 1410, Mbarara, Uganda; 5 Directorate of Graduate Studies, Research and Innovations, Busitema University, P.O. Box 1460, Busia, Uganda; 6 Department of Pathology, Busitema University, Faculty of Health Sciences, P.O. Box 1460, Mbale, Uganda

**Keywords:** Alcohol, Substance use, University, Students, Uganda, Psychology, Medical research

## Abstract

Use of alcohol and other substances remains a major health concern among higher learning institutions. This study aimed at assessing the prevalence of alcohol and other substance use among students at Busitema University in Eastern Uganda. A cross sectional survey was conducted among 658 undergraduate students using a questionnaire consisting of Alcohol, Smoking and Substance Involvement Screening Tool and participant sociodemographic and clinical factors. Logistic regression was used to explore the associations. Two hundred sixty-five (40.3%) students reported ever using alcohol and 158 (24.0%) had used in last 3 months. Seventy-four (11.2%) students reported ever use of other substances including tobacco, cannabis, cocaine, stimulants sedatives and hallucinogens and 36 (5.5%) had used within the recent 3 months. After controlling for potential confounders, recent alcohol use was associated with engaging in romantic relationship (odd ratio (OR) = 1.9,* P* value (*P*) = 0.045) while having chronic medical conditions was protective (OR = 0.3, *P* = 0.031). On the other hand, recent use of other substances was 7 times higher among males (OR = 7.0, *P* = 0.008) compared to females while fourth year of study was protective (OR = 0.05, *P* = 0.011). Although alcohol use is a worsening challenge among university students, use of other substances is also highly prevalent after COVID-19 lockdown. There is need for universities to identify students with above factors and design interventions to address them in order to prevent the likely undesirable outcomes of alcohol and substance use.

## Introduction

Globally, the burden of annual alcohol consumption per person aged 15 years and above is 6.5 L of pure alcohol. Additionally, 3.5–5.7% of the world’s population reported consuming at least one other psychoactive substances including cannabis, opioids, injection drugs, stimulants among other respectively^1^. In 2020, alcohol accounted for 1.78 million deaths globally and about 59% of people aged 15–39 years were engaging in harmful drinking^2^. In low and middle income countries the prevalence of these substances varies widely ranging from 5.8% for alcohol in Sub-Saharan Africa^3^, 5.2–13.5% for cannabis and 3.7% for injection drugs in West and Central Africa^1^.

In 2018, the per capita alcohol consumption in Uganda was 15.1 among persons aged 15 years and above^3^. A nation-wide survey indicated that alcohol is the commonest substance with current use among adult persons was 26.8% with 9.8% having an alcohol-use-related disorder and majority (76.9%) being aged between 18 and 49 years^4^. Other substances that also reported to be commonly used by young people at educational institutions in Uganda include tobacco, stimulants and opioids inhalants among others^5^. In Uganda, most students join University at the age of 18 years and above which is the age at highest risk of substance use^6^. Existing studies indicate that prevalence of substance use among university students varies widely ranging from 31% for alcohol^7^, cannabis at 8% and tobacco at 7%^8^.

Whereas substances other than alcohol are considered to be harmful and hence illegal in most countries, World Health Organisation also advises that no amount of alcohol is good for one’s health hence should be avoided^9^. However, there are various factors that influence substance use and they vary geographically due to the diverse sociocultural factors and also as per substance^2,10,11^. For instance in Egypt substance use was found to be significantly associated with being male and attending practical college^12^. In Yemen and Saudi Arabia, there was statistically significant difference in prevalence of use of substances like prescription drugs such as stimulants and sedatives and was found to be higher among males compared to females^13^.

Some studies have documented that alcohol and other substance use has been associated with having mental illness like depression and anxiety disorders^14^, excessive academic stress^7^ and type of residence at university^8^. Use of substances such as cannabis have been associated with academic difficulties among students characterized by skipping classes and poor academic performances^15^. In Kerala India, tobacco use among college students was mainly associated with male gender and comorbid use of alcohol^16^. Similarly hallucinogen use among university students has been associated with comorbid use of other substances like alcohol as well as have mental health problems, risky sexual behaviors, low self-esteem and impulsivity personality traits^17^. Among college students in the United States, engaging in romantic relationship was highly associated with binge alcohol drinking, marijuana and nicotine use but not prescription drugs^18^.

At a time when most countries including Uganda are recovering from COVID-19 pandemic and its related lockdown, substance use is likely to be on the rise as some people resorted to it as a coping mechanism. Hence, University students who underwent restrictions such as suspension of academic activities, transition to online versus physical studying and other psychosocial change were at higher risk of using substances to deal with depressive and anxiety symptoms^19^. These post-COVID effects come as additions to the already existing academic, social and financial challenges at University.

Unfortunately, literature regarding the status of alcohol and related substance use among university students after COVID-19 pandemic remains scanty. Whereas some studies have been conducted during and post-COVID-19 lockdown to assess substance use among students of higher institutions of learning and the finding vary geographically^20–23^. Hence this calls for context specific research to inform local policies and interventions. Therefore, this study aimed at determining the prevalence of alcohol and other related substance use among undergraduate students at Busitema University in rural Eastern Uganda. By understanding the burden and determinants of substance use, we can be able to design context specific intervention geared towards reduction of the burden of use and resultant complication for a more productive university population.

## Methods and materials

### Study design and site

This was a cross sectional survey conducted at Busitema University (BU) in Eastern Uganda. BU is a multi-campus public university having six campuses spread across 6 districts in this region with each offering different courses. This study was conducted at the main (Busitema) campus which is in Tororo district and Mbale campus which is in Mbale district. Busitema campus offers mainly engineering courses like civil and electrical engineering at certificate, diploma and bachelor’s degree levels while Mbale campus offers medical courses such as medicine and surgery, nursing, and anesthesia at bachelor’s degree level. The study was conducted at these two sites due to proximity and comparability since they all offer science related courses. Additionally, Busitema is the main campus for the University while most cases of mental illness had been reported at Mbale campus prior to conducting this study^24,25^. At the time of conducting the study, Busitema campus had approximately 700 students while Mbale campus had about 490 students.

### Study population, sampling and recruitment

Eligible participants included any undergraduate students aged above 18 years attending any of the above two campuses who were available at the time of data collection. Students having any severe mental or physical illness that could make them unable to respond meaningfully to research questions were excluded. Participants were recruited at their respective campuses during their free time or at times when they had breaks from academic activities to minimize interference with academic work. These were approached by trained research assistants who would explain the purpose, benefits and risks of the study and hence obtain written informed consent prior to participation. Each participant was required to present a valid university identity card and would further be verified by checking on the respective student list obtained from university administration for each campus.

A total of 658 students (298 and 360 students at Mbale and Busitema campuses respectively) were recruited and this was estimated using Cochran (1977) formula^26,27^ considering two clusters (two campuses), Inter-Cluster Correlation of 0.5 between two campuses, design effect of 1.5, alpha of 0.05 and z value of 1.96 at 95% confidence level and 10% non-response rate. The prevalence of substance use disorders was considered to be 50% in order to achieve the maximum sample size. Sample sizes at each campus were based on the proportion of the respective total population of students. Stratified sampling was used based on the eligibility criteria in relation to both sites. At each site, proportions of students to be recruited were determined by the relative total number of students in each year of study. For each year of study, participants were recruited consecutively until the required sample sizes were achieved at both sites.

### Data collection and management

Data was collected by well-trained research assistants using an electronic questionnaire installed on tablets designed using Google form software. The questionnaire consisted of mainly three sections: (1) sociodemographic characteristics, (2) social and clinical factors influencing substance use, and (3) Alcohol, Smoking and Substance Involvement Tool (ASSIST). ASSIST is used for screening alcohol and other psychoactive substances including tobacco products, cannabis, cocaine, amphetamine-type stimulants, sedatives and sleeping pills, hallucinogens, inhalants, opioids, injection drugs and others. It was developed by World Health Organisation for primary health care and community settings. It has eight items with question 1 and 8 assessing “ever use” while questions 2–7 assess substance use in the last 3 months. Each item is scored on a likert scale with responses such as “never” = 0, “once or twice” = 2, “monthly” = 3, “weekly” = 4, “daily/almost daily” = 6 (for question 2). However, these assigned numerical scores differ from question to question and the total risk score is calculated by adding score of questions 2 to 7 with the minimum being 0 and maximum is 39. The total score can be categorized into the ASSIST risk score whereby for alcohol, lower risk ranges from 0–10, moderate risk is from 11–26, and high risk = 27 and above while for other substances, lower risk ranges from 0–3, moderate risk is from 4–26 and high risk is 27 and above^28^. This tool has been validated and used in different settings including Uganda and has high reliability with sensitivity ranging from 65 to 75% and specificity of 69–80% respectively depending on the substance and ASSIST risk score^29–31^.

For this study, the main outcome variables were alcohol use and other substance use as assessed by ASSIST. The independent variables included sociodemographic and clinical factors influencing substance used as derived from existing literature like age, sex, socioeconomic status, academic-related stressors, personal and familial history of mental or physical illnesses, among others^12,30^ (see Appendix 1).

Each participant was assigned a unique identification number for this study and completed forms were submitted to an online server which was password protected and only accessible by or with authorization for principal investigator. The completed forms were downloaded in an excel format, checked for completeness, cleaned, and coded before analysis.

### Data analysis

The final excel sheet was imported into STATA version 16 software for analysis. Summary statistics were calculated, whereby for dichotomous or categorical variables frequencies and percentages were reported while for continuous variables, means and corresponding standard deviations were reported. Prevalences of alcohol and other substance use were calculated as proportions of participants scoring above set cutoff points on ASSIST. Any student who responded “Yes” (score 3) for any specific substance as per question 1 was considered as having ever used that substance. Also, any student who scored 2 and above on question 2 was considered as having used that specific substance in the last 3 months. Factors associated with alcohol use and use of other substances were determined using bivariable and multivariate logistic regression. The measure of association was odds ratio considering 95% confidence interval and statistical significance of less than 5%. Variables with a *P* value of 0.2 at bivariable analysis were included in the multivariate logistic regression model.

### Quality control

The study protocol was developed based on existing high quality scientific literature and was followed strictly and carefully throughout the study. The most senior team member was the quality control coordinator throughout the study. Research assistants completed a course in Good Clinical Practice and protection of human participants and were trained in the administration of data collection tools such as ASSIST. All study tools administered in English for uniformity by all research assistants. During data collection, the principal investigator would routinely sit in interview sessions by the different research assistants to assess their performance and provide feedback and guidance whenever necessary. Data collection software was designed with checks that minimized errors or missing data. Data collection tools were pilot tested among university students for standardization. Data analysis software will also be programmed to flag missing data, out of range or illogical values during analysis.

### Ethical considerations

Ethical approval was sought from Busitema University Faculty of Health Sciences Research Ethics Committee (Number: BUFHS-2022-11) and Uganda National Council of Science and Technology (Number: HS2700ES). Permission to collect data at the respective study sites was sought from the University administration and each student provided written informed consent prior to participation in the study. All data collection, management and analysis procedures were conducted according to guidelines and regulations by the Uganda National Council of Science and Technology.

## Results

### Participant characteristics and prevalence of substance use

**Figure 1:**
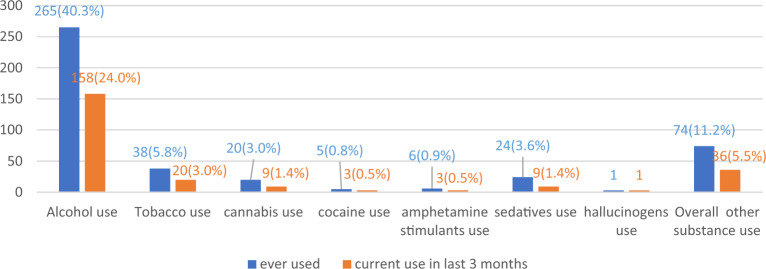
Graph showing prevalence of common substances used by students.

Majority of the participants were males (63.7%), born again Christians (44.5%) and single (83.4%). Over 67% of the students were privately sponsored, 54.3% were coming from semi-urban homes, and 62.2% were taking with maximum duration of 4 years. Tables 1 and 2 show the distribution of the various characteristics of the study participants that had either alcohol or other substance use within the last 3 months to the study. There proportion of students engaging in romantic relationships (*P* = 0.027) and having dependents (*P* = 0.038) who used alcohol in the last 3 months was significantly higher than those that did not. Also, there was a probable association between year of study (*P* = 0.019) and use of other substances in the last 3 months. Out of the 658 participants, 265 (40.3%) and 74 (11.2%) reported history of ever use of alcohol and other substances respectively (see Fig. 1).

**Table 1:** Characteristics of study participants with history of alcohol or other substance use within the last 3 months. Significant values are in bold.

Variables	Alcohol use, n (%)				Other substance use, n (%)			
	Total ever use n = 265 (%)	Use within last 3 months			Total ever use n = 74 (%)	Use within last 3 months		
		No n = 107 (%)	Yes n = 105 (%)	*P*-value		No n = 38 (%)	Yes n = 36 (%)	*P*-value
Age (years)				0.764				0.153
18–24	178 (67.2)	73 (68.2)	105 (66.5)		54 (73.0)	25 (65.8)	29 (80.6)	
> = 25	87 (32.8)	34 (31.8)	53 (33.5)		20 (27.0)	13 (34.2)	7 (19.4)	
Sex				0.185				0.097
Female	82 (30.9)	38 (35.5)	44 (27.8)		21 (28.4)	14 (36.8)	7 (19.4)	
Male	183 (69.1)	69 (64.5)	114 (72.2)		53 (71.6)	24 (63.2)	29 (80.6)	
Religion				0.977				0.564
Anglican	73 (27.5)	30 (28.0)	43 (27.2)		17 (23.0)	9 (23.7)	8 (22.2)	
Catholic	76 (28.7)	28 (26.2)	48 (30.4)		17 (23.0)	11 (28.9)	6 (16.7)	
Born-again Christian	99 (37.4)	42 (39.3)	57 (36.1)		33 (44.6)	14 (36.8)	19 (52.8)	
SDA	10 (3.8)	4 (3.7)	6 (3.8)		3 (4.1)	1 (2.6)	2 (5.6)	
Muslim	3 (1.1)	1 (0.9)	2 (1.3)		1 (1.4)	1 (2.6)	0 (0.0)	
Others	4 (1.5)	2 (1.9)	2 (1.3)		3 (4.1)	2 (5.3)	1 (2.8)	
Marital status				0.266				0.761
Married	28 (10.6)	14 (13.1)	14 (8.9)		7 (9.5)	3 (7.9)	4 (11.1)	
Single	221 (83.4)	89 (83.2)	132 (83.5)		62 (83.8)	33 (86.8)	29 (80.6)	
Cohabiting	16 (6.0)	4 (3.7)	12 (7.6)		5 (6.8)	2 (5.3)	3 (8.3)	
Faculty				0.608				0.658
Health sciences	114 (43.0)	44 (41.1)	70 (44.3)		33 (44.6)	16 (42.1)	17 (47.2)	
Engineering	151 (57.0)	63 (58.9)	88 (55.7)		41 (55.4)	22 (57.9)	19 (52.8)	
Year of study				0.239				**0.019**
1	111 (41.9)	39 (36.4)	72 (45.6)		29 (39.2)	15 (39.5)	14 (38.9)	
2	51 (19.2)	23 (21.5)	28 (17.7)		11 (14.9)	3 (7.9)	8 (22.2)	
3	40 (15.1)	21 (19.6)	19 (12.0)		17 (23.0)	6 (15.8)	11 (30.6)	
4	56 (21.1)	20 (18.7)	36 (22.8)		15 (20.3)	13 (34.2)	2 (5.6)	
5	7 (2.6)	4 (3.7)	3 (1.9)		2 (2.7)	1 (2.6)	1 (2.8)	
Source of funding				0.978				0.680
Private	186 (70.2)	75 (70.1)	111 (70.3)		49 (66.2)	26 (68.4)	23 (63.9)	
Government	79 (29.8)	32 (29.9)	47 (29.7)		25 (33.8)	12 (31.6)	13 (36.1)	
University residence				0.965				0.977
Private (self)	140 (52.8)	57 (53.3)	83 (52.5)		32 (43.2)	16 (42.1)	16 (44.4)	
University hall	122 (46.0)	49 (45.8)	73 (46.2)		40 (54.1)	21 (55.3)	19 (52.8)	
Home (with guardian)	3 (1.1)	1 (0.9)	2 (1.3)		2 (2.7)	1 (2.6)	1 (2.8)	
Home residence				0.304				0.230
Rural	41 (15.5)	21 (19.6)	20 (12.7)		9 (12.2)	7 (18.4)	2 (5.6)	
Semi urban	137 (51.7)	53 (49.5)	84 (53.2)		43 (58.1)	20 (52.6)	23 (63.9)	
Urban (city)	87 (32.8)	33 (30.8)	54 (34.2)		22 (29.7)	11 (28.9)	11 (30.6)	
Region of origin				0.292				0.724
Eastern	116 (43.8)	53 (49.5)	63 (39.9)		34 (45.9)	18 (47.4)	16 (44.4)	
Western	52 (19.6)	18 (16.8)	34 (21.5)		14 (18.9)	7 (18.4)	7 (19.4)	
Central	49 (18.5)	20 (18.7)	29 (18.4)		15 (20.3)	6 (15.8)	9 (25.0)	
Northern	47 (17.7)	15 (14.0)	32 (20.3)		10 (13.5)	6 (15.8)	4 (11.1)	
Non-Ugandan	1 (0.4)	1 (0.9)	0 (0.0)		1 (1.4)	1 (2.6)	0 (0.0)	
Family financial status				0.303				0.917
Quite well off	133 (50.2)	59 (55.1)	74 (46.8)		37 (50.0)	19 (50.0)	18 (50.0)	
Not well off	109 (41.1)	37 (34.6)	72 (45.6)		32 (43.2)	16 (42.1)	16 (44.4)	
Wealthy	7 (2.6)	4 (3.7)	3 (1.9)					
Poor	16 (6.0)	7 (6.5)	9 (5.7)		5 (6.8)	3 (7.9)	2 (5.6)	
Maximum duration of course				0.710				0.554
0.5	1 (0.4)	1 (0.9)	0 (0.0)					
2	18 (6.8)	7 (6.5)	11 (7.0)		3 (4.1)	2 (5.3)	1 (2.8)	
4	165 (62.3)	68 (63.6)	97 (61.4)		48 (64.9)	23 (60.5)	25 (69.4)	
5	79 (29.8)	31 (29.0)	48 (30.4)		22 (29.7)	13 (34.2)	9 (25.0)	
6	1 (0.4)	0 (0.0)	1 (0.6)					
7	1 (0.4)	0 (0.0)	1 (0.6)		1 (1.4)	0 (0.0)	1 (2.8)

**Table 2:** Characteristics of study participants with history of alcohol or other substance use within the last 3 months. Significant values are in bold.

Variables	Alcohol use, n (%)				Other substance use, n (%)			
	Total ever use n = 265 (%)	Use within the last 3 months			Total ever use n = 74 (%)	Use within the last 3 months		
		No n = 107 (%)	Yes n = 105 (%)	*P*-value		No n = 38 (%)	Yes n = 36 (%)	*P*-value
Had a retake (yes)	8 (3.0)	1 (0.9)	7 (4.4)	0.103	3 (4.1)	1 (2.6)	2 (5.6)	0.524
History of chronic medical condition	21 (7.9)	12 (11.2)	9 (5.7)	0.103	9 (12.2)	2 (5.3)	7 (19.4)	0.062
Often worried about academic performance	145 (54.7)	59 (55.1)	86 (54.4)	0.909	48 (64.9)	24 (63.2)	24 (66.7)	0.752
Often worried about academic activities	89 (33.6)	31 (29.0)	58 (36.7)	0.191	36 (48.6)	15 (39.5)	21 (58.3)	0.105
Bullied by students	24 (9.1)	9 (8.4)	15 (9.5)	0.763	7 (9.5)	3 (7.9)	4 (11.1)	0.637
Bullied by teachers	26 (9.8)	11 (10.3)	15 (9.5)	0.833	11 (14.9)	8 (21.1)	3 (8.3)	0.124
Involved in romantic relationship	213 (80.4)	79 (73.8)	134 (84.8)	**0.027**	58 (78.4)	30 (78.9)	28 (77.8)	0.903
Feel pressured by relatives about your academics	96 (36.2)	38 (35.5)	58 (36.7)	0.843	38 (51.4)	18 (47.4)	20 (55.6)	0.481
Have dependents	62 (23.4)	18 (16.8)	44 (27.8)	**0.038**	16 (21.6)	8 (21.1)	8 (22.2)	0.903
Family history of mental illness	55 (20.8)	17 (15.9)	38 (24.1)	0.108	18 (24.3)	8 (21.1)	10 (27.8)	0.500
Family history of chronic medical illness	150 (56.6)	58 (54.2)	92 (58.2)	0.517	41 (55.4)	18 (47.4)	23 (63.9)	0.153
Choose by yourself to undertake course of study	227 (85.7)	95 (88.8)	132 (83.5)	0.232	56 (75.7)	28 (73.7)	28 (77.8)	0.682
Assured of getting tuition or upkeep	194 (73.2)	82 (76.6)	112 (70.9)	0.300	56 (75.7)	26 (68.4)	30 (83.3)	0.135

Also, alcohol was the most prevalent substance used within the last 3 months at 24% while use of other substances was at 5.5% with tobacco being the most used at 3% (see Fig. 1). Notably, no student reported ever use of opioids or any other injection drugs at both campuses.

Considering participants who had ever used alcohol or any other substance, moderate to high-risk use was greater among participants using other substances (21.4%) compared to alcohol (12.9%). (see Table 3).

**Table 3:** ASSIST risk score for alcohol and other substance use among Busitema University students.

Variables	N	Severity of alcohol and other substance use		
		Low risk	Moderate risk	High risk
Alcohol use	265	231 (87.2)	33 (12.5)	1 (0.4)
Other substance use				
Tobacco use	38	33 (86.8)	5 (13.2)	0 (0.0)
Cannabis use	20	15 (75.0)	5 (25.0)	0 (0.0)
Cocaine use	5	3 (60.0)	2 (40.0)	0 (0.0)
Amphetamine stimulants use	6	4 (66.7)	2 (33.3)	0 (0.0)
Sedatives use	24	21 (87.5)	3 (12.5)	0 (0.0)
Hallucinogens use	1	1 (100.0)	0 (0.0)	0 (0.0)
Overall other substance use	74	58 (78.4)	15 (20.3)	1 (1.4)

Bivariate and multivariate analysis using logistic regression showed that students with chronic medical conditions were less likely to use alcohol within last 3 months (odd ratio (OR) = 0.3, *P* value = 0.031). Conversely, students involved in a romantic relationship were almost twice more likely (OR = 1.9,* P* value = 0.045) to use alcohol within the last 3 months compared to those not involved in such relationship. (see Table 4).

**Table 4:** Factors associated with alcohol use within the last 3 months among students (n = 265). Significant values are in bold.

Variables	Crude OR (95% CI)	*P* value	Adjusted OR (95% CI)	*P* value
Sex				
Female	1		*1*	
Male	1.4 (0.8, 2.4)	0.186	1.7 (0.9, 2.9)	0.076
Had a retake	4.9 (0.6, 40.5)	0.139	3.5 (0.4, 31.8)	0.263
History of chronic medical condition	0.5 (0.2, 1.2)	0.109	0.3 (0.1, 0.9)	**0.031**
Often worried about academic activities	1.4 (0.8, 2.4)	0.192	1.4 (0.8, 2.5)	0.227
Involved in romantic relationship	2.0 (1.1, 3.6)	0.029	1.9 (1.02, 3.7)	**0.045**
Have dependents	1.9 (1.03, 3.5)	0.039	1.7 (0.9, 3.3)	0.117

Regarding use of other substances within the last 3 months, the odds were 7 times higher among males (OR = 7.0, *P* = 0.008) compared to females while for being in fourth year of study was protective (OR = 0.05, *P* value = 0.011) against use compared to other years (see Table 5).

**Table 5:** Factors associated with other substance use within the last 3 months among students (n = 74). Significant values are in bold.

Variables	Crude OR (95% CI)	*P*-value	Adjusted OR (95% CI)	*P*-value
Age (years)				
18–24	1		1	
> = 24	0.5 (0.2, 1.3)	0.157	0.3 (0.1, 1.5)	0.166
Sex				
Female	1		1	
Male	2.4 (0.8, 6.9)	0.102	7.0 (1.7, 30.3)	**0.008**
Year of study				
1	1		1	
2	2.9 (0.6, 13.0)	0.174	3.3 (0.6, 19.1)	0.190
3	2.0 (0.6, 6.7)	0.283	2.3 (0.5, 10.1)	0.267
4	0.2 (0.03, 0.9)	0.033	0.05 (0.04, 0.5)	**0.011**
5	1.1 (0.1, 18.8)	0.962	1.4 (0.04, 45.6)	0.859
History of chronic medical condition	4.3 (0.8, 22.5)	0.080	11.5 (0.9, 152.6)	0.063
Often worried about academic activities	2.1 (0.8, 5.4)	0.107	2.6 (0.7, 9.7)	0.161
Family history chronic medical illness	2.0 (0.8, 5.0)	0.155	2.4 (0.6, 8.6)	0.197
Assured of getting tuition upkeep	2.3 (0.8, 7.0)	0.140	0.9 (0.2, 4.3)	0.888

## Discussion

This study aimed at determining the prevalence and associated factors of alcohol and other substance use among undergraduate students at Busitema University. The findings indicated that prevalence of ever use and use within last 3 months of alcohol use was 40.3% and 24.0% while for other substances was 11.2% and 5.5% respectively. Risky use of alcohol and other substances was higher among male than female students. Current alcohol use among students was associated with being involved in a romantic relationship while having chronic medical conditions was protective while use of other substances was associated with being male while fourth year of study was protective.

These findings are in line with studies that have documented similar prevalence of alcohol use among university students. For example at Makerere University prevalence of alcohol use was 39% among social media users though this was in a 12-month period and use in last 3 months was never assessed^32^. However, our findings differed from what was reported among undergraduate students at Gulu University of 35%^33^ and 52.9% at Mbarara University^34^. This difference may be explained by differences in study tools used for example the former used Alcohol Use Disorder Identification Test which assesses alcohol use disorder^33^ while the latter^34^ used no standard tool for assessing alcohol use and none of these assessed ever use or use within last 3 months. Additionally, the differences in prevalences could be due to geographical variations whereby the above two institutions are in northern and southwestern regions respectively hence having students who predominantly come from those regions. This study was conducted at Busitema and Mbale campuses of Buistema University which mainly serves eastern Uganda. The geographical distribution of these Universities is associated with social cultural differences which greatly influence substance use behaviors among people in the specific regions.

However, a study conducted among undergraduate students in Kenya using ASSIST indicated that 21.9% of students ever used alcohol while only 16.9% had used within the recent 3 months^8^ which are markedly lower than our findings. Hence this indicates that the problem is higher in Eastern Uganda which may be one of the effects of COVID-19 since the Kenya study was conducted before COVID-19 pandemic.

Additionally, the prevalence of other substance use in this study was higher than what has been documented among the same Kenyan student population whereby prevalence of ever use of any other substance was 9.4% with cannabis being the commonest rather than tobacco as per our setting^8^.

Conversely, studies in other settings have reported varying prevalence of use of other substances among university students such as 8.9% for tobacco and 4.3% for sedatives in Egypt^35^, 41% for Khat (amphetamine), 22% for Cigarettes and 7.4% for illicit drugs in Ethiopia^36^ and 52% for cannabis, 25% for cocaine and 9% for amphetamines in Ireland^37^. These variations in prevalence of use of different substances are influenced by psychosocial and cultural factors within the different contexts hence suggesting need for context-specific studies and interventions.

Generally, there was a higher prevalence of alcohol use among university students compared to other substances. This could be because alcohol is legally available to all persons aged 18 years and above as per the current regulatory policies. It is also readily available and affordable on the market in various forms with some types being locally brewed in homes which exposes students to its use early in life. Additionally, most sociocultural groups in the eastern region consider alcohol use to be a culturally acceptable practice and some homes sell it as source of livelihood hence students from such backgrounds are prone to using it^38^. However, some are able to break the chain by cessation of drinking as they grow hence explaining the lower prevalence of current use compared to ever use.

Unlike alcohol, most of the other substances such as cannabis, cocaine and others are illegal and not readily available for sale on market which makes accessibility more difficult which possibly explains the lower prevalence of both ever and current use^39^. This is in line with finding from most of the existing literature which has documented alcohol to be the most commonly used substance among students^40,41^. Generally, more students had low risk use of alcohol and other substances which show the need to ensure that they keep abstaining or do not worsen their substance use habits to prevent progression to high-risk use. However, for those with moderate to high risk use (especially those using other substances), more intensive interventions to counteract their current drinking, smoking and other substance use habits^42^.

Recent alcohol use was found to be significantly associated with being involved in a romantic relationship which may be more of a poor coping mechanism used by students due to social pressures resulting from such relationships. Recent studies have indicated that Ugandan university students engage in sexual relationships referred to as “situationships” with different individuals including their peers, teachers and other people mainly to navigate socioeconomic challenges or for monetary purposes^43^. Hence, alcohol use may be related to students trying to boost their confidence to face challenges resulting from such relationships like rejection, separation, or any other form of disappointments. For other students especially the youth, this may be due to peer pressure or role modelling effect from their partners who use substances compelling them to start use in order to fit in the group^44^. Notably, partner influence has been found to be strongly associated with alcohol use with females having more influence than males^45^. Whereas this influence can be protective, sometimes in this case it may be responsible for the high rates of substance use among the couples. Hence this may mean that many students are currently using alcohol because of influence by their romantic relationship partners.

Conversely, students having chronic medical conditions were less likely to use alcohol which may be because of the fear of worsening their pre-existing medical illnesses. Also, by having chronic medical illnesses these students are more likely to receive medical education and counselling discouraging them from using alcohol in order not to exacerbate their illnesses. This is consistent with findings from primary healthcare settings in California where people with medical conditions were less likely to drink alcohol^46^. However, it is important to consider that chronic alcohol use has also been associated with many other chronic medical conditions such as hypertension, diabetes, liver disease and others, though these are less common in our study population^47^.

Unlike alcohol, use of other substances was associated with being male which could be explained by the aggressive nature of males compared to females which makes them able to search and obtain these illegal substances. This is in line with what was reported in other studies where males were more likely to use substances compared to females^8,12,33^. Commonly, males are more likely to have antisocial, extraversion and impulsive personality traits which are more associated with risky substance use and hence several studies have found higher prevalence of illicit drug use among them compared to females^48–51^.

On the other hand, fourth year of study was protective against use of other substances. Since this is the final year for most courses offered at both campuses, these students are usually more likely to be more hopeful in the life after school where they are expected to act professionally hence reducing their chances of substance use. Relative to other years of study, where there may be more stressor including some from teachers, final year students tend to be kindlier by their teachers who may become colleagues after their completion hence the protective effect towards substance use.

### Implications of study findings

The findings indicate that whereas alcohol remains the commonest substance used by students at the university, other substances like tobacco, cannabis, cocaine, amphetamines, sedatives and hallucinogens are also in use. Hence interventions and education and health policies should broaden their scope to curb the use of all these substances. The factors associated with alcohol and other substance use are both non-modifiable indicating that intervention strategies should mainly aim at preventive measure involving screening for the above risk factors among students while reinforcing the protective ones.

### Study limitations

The study was conducted at only two out of six campuses of Busitema University hence the findings may not perfectly reflect the exact situation at other campuses or even other universities. However, the findings shed light on what is going on at the university in relation to substance use among students and hence calling for possible interventions. Since the study investigated a sensitive social issue, there might have been response bias from some participants especially regarding use of illegal substances. This was minimized by using independent well trained research assistants within the age range of most students who were not university staffs to enhance freeness during interviews.

## Conclusion and recommendations

Like findings from other universities in Uganda, use of alcohol and other substance remains a significant health challenge among undergraduate students at Busitema Universities especially at a time when the country is struggling with effects of COVID-19 pandemic. The factors associated with use of these substances occur both at individual and institutional level. This calls for interventions at national and institutional levels for-example ensuring health relationships among students or regulating the marketing and acquisition of some of the currently legal products such as alcohol and tobacco by students. Institutions may also need to put in place measures to monitor any involvement in transactions leading to acquisition or use of any substances in institutions. Finally, there is need for more research regarding measures or interventions to manage and rehabilitate students who may have any substance use disorders to ensure that they successfully study and complete their respective courses.

### Supplementary Information

Supplementary Information.

## Data Availability

The datasets used and/or analysed during the current study are available from the corresponding author on reasonable request.
