# Supplementary material for: Burden of alcohol and other substance use and correlates among undergraduate students at Busitema University in rural Eastern Uganda after COVID-19 lockdown
Source: Sci Rep. 2024 Mar 14;14:6194. doi: 10.1038/s41598-024-56861-1 (PMC10940298; doi:10.1038/s41598-024-56861-1)
Supplement: Supplementary file 1 — Supplementary Information. [file 41598_2024_56861_MOESM1_ESM.docx]

1. **PARTICIPANT SOCIODEMOGRAPHICS AND CLINICAL FACTORS**

Age (in years):

Sex: Male =0 Female =1 Prefer not to say =2

Religion: Anglican=0 catholic=1 born again Christian =2 SDA = 3 Muslim = 4 others (specify) = 5

Marital status: single=0 married =1 cohabiting =2 others (specify) =3

Faculty: **Engineering** =0 **Health Sciences** =1

Year of study: one =0 two =1 three =2 four =3 five =4

Source of funding: Government =0 Private = 1

University Residence: University hall =0 Home (with guardian) =1 private(self) =2 others (specify) =3

Home residence: Urban (city) =0 semi-urban =1 Rural =2

Region of origin: Eastern =0 western =1 Northern =2 central =3 non-Ugandan =4

Considering your family background, how would you describe your financial status?

Wealthy =0 quite well off =1 not well off =2 poor =3

What is the maximum duration of your course (in years)? …..

Have you repeated any year/course unit (by retake)? Yes =1 No=0

If yes, how many years?

Do you have any known history of a chronic or longstanding medical condition? Yes =1 No =0

If yes, specify…

Do you often feel worried about your academic performance? Yes =1 No =0

Do you often feel worried about attending lectures, tutorial or any other academic activities? Yes =1 No =0

Were you bullied or are you being bullied by fellow students at university? Yes =1 No =0

Were you bullied or are you being bullied by teachers/lecturers? Yes =1 No =0

Have you been involved in a romantic relationship? Yes =1 No =0

Do you feel pressured by your family/relatives about your academics? Yes =1 No =0

Do you have any dependents? Yes =1 No =0.

Do you have any relative or family member having known chronic mental illness? Yes =1 No =0

If yes, specify

Do you have any relative or family member having a known chronic medical illness? Yes =1 No =0

Did you choose by yourself to undertake the current course of study? Yes =1 No =0

Are you assured of getting tuition or upkeep? Yes =1 No =0

Thank you for your time.
